# Schistosomiasis-associated pulmonary hypertension unveils disrupted murine gut–lung microbiome and reduced endoprotective Caveolin-1/BMPR2 expression

**DOI:** 10.3389/fimmu.2023.1254762

**Published:** 2023-10-16

**Authors:** Ygor Marinho, Elizabeth S. Villarreal, Sammy Y. Aboagye, David L. Williams, Jun Sun, Claudia L. M. Silva, Sarah E. Lutz, Suellen D. Oliveira

**Affiliations:** ^1^ Vascular Immunobiology Lab, Department of Anesthesiology, College of Medicine, University of Illinois Chicago, Chicago, IL, United States; ^2^ Department of Microbial Pathogens and Immunity, Rush University Medical Center, Chicago, IL, United States; ^3^ Department of Medicine, College of Medicine, University of Illinois Chicago, Chicago, IL, United States; ^4^ Molecular and Biochemical Pharmacology Lab, Institute of Biomedical Sciences, Federal University of Rio de Janeiro, Rio de Janeiro, RJ, Brazil; ^5^ Department of Anatomy and Cell Biology, College of Medicine, University of Illinois Chicago, Chicago, IL, United States; ^6^ Vascular Immunobiology Lab, Department of Physiology and Biophysics, College of Medicine, University of Illinois Chicago, Chicago, IL, United States

**Keywords:** schistosomiasis, endothelial cell, PAH, apoptosis, Cav-1, BMPR2, lung microbiome, gut microbiome

## Abstract

Schistosomiasis-associated Pulmonary Arterial Hypertension (Sch-PAH) is a life-threatening complication of chronic *S. mansoni* infection that can lead to heart failure and death. During PAH, the expansion of apoptosis-resistant endothelial cells (ECs) has been extensively reported; however, therapeutic approaches to prevent the progression or reversal of this pathological phenotype remain clinically challenging. Previously, we showed that depletion of the anti-apoptotic protein Caveolin-1 (Cav-1) by shedding extracellular vesicles contributes to shifting endoprotective bone morphogenetic protein receptor 2 (BMPR2) towards transforming growth factor beta (TGF-β)-mediated survival of an abnormal EC phenotype. However, the mechanism underlying the reduced endoprotection in PAH remains unclear. Interestingly, recent findings indicate that, similar to the gut, healthy human lungs are populated by diverse microbiota, and their composition depends significantly on intrinsic and extrinsic host factors, including infection. Despite the current knowledge that the disruption of the gut microbiome contributes to the development of PAH, the role of the lung microbiome remains unclear. Thus, using a preclinical animal model of Sch-PAH, we tested whether *S. mansoni* infection alters the gut–lung microbiome composition and causes EC injury, initiating the expansion of an abnormal EC phenotype observed in PAH. Indeed, *in vivo* stimulation with *S. mansoni* eggs significantly altered the gut–lung microbiome profile, in addition to promoting injury to the lung vasculature, characterized by increased apoptotic markers and loss of endoprotective expression of lung Cav-1 and BMPR2. Moreover, *S. mansoni* egg stimulus induced severe pulmonary vascular remodeling, leading to elevated right ventricular systolic pressure and hypertrophy, characteristic of PAH. *In vitro*, exposure to the immunodominant *S. mansoni* egg antigen p40 activated TLR4/CD14-mediated transient phosphorylation of Cav-1 at Tyr14 in human lung microvascular EC (HMVEC-L), culminating in a mild reduction of Cav-1 expression, but failed to promote death and shedding of extracellular vesicles observed *in vivo*. Altogether, these data suggest that disruption of the host-associated gut–lung microbiota may be essential for the emergence and expansion of the abnormal lung endothelial phenotype observed in PAH, in addition to *S. mansoni* eggs and antigens.

## 1 Introduction

Globally, more than 200 million people are currently infected by intravascular parasites from the genus *Schistosoma*, *and* approximately 10 million develop pulmonary arterial hypertension (Sch-PAH), indicating that the disease is the leading cause of PAH worldwide (1). Sch-PAH is a life-threatening complication of chronic *Schistosoma* infection, including *S. mansoni*. Sch-PAH shares similar features with idiopathic PAH (IPAH, unknown cause), including TGF-β-mediated vascular remodeling (2, 3). The disease is characterized by obliteration and remodeling of the pulmonary vasculature in response to *S. mansoni* egg translocation from the mesenteric system (4). Then, the remodeled and occluded pulmonary vasculature increases vascular resistance, leading to right ventricular hypertrophy (RVH), heart failure, and premature death. Currently, there are no targeted therapies for Sch-PAH (1).

After infection, *S. mansoni* migrates through the cardiovascular system, reaching the mesenteric circulation where the parasite lays its eggs (4). Within the mesentery, the eggs cross the intestinal wall, disturbing the gut microbiome or migrating to other organs, including the lungs, where they can lead to PAH. Recent findings indicate that similar to the gut, the lungs are populated by a diverse microbiota (5), but it was unclear whether its disruption (i.e., dysbiosis) also contributed to the development of PAH. It is clear that inside the host vasculature, *S. mansoni* and their eggs interact directly with the endothelium in multiple organs, including the lungs. In this process, ECs are not simple bystanders; they are also highly efficient in killing the immature form of *S. mansoni* (6), and their interaction with the parasite eggs significantly contributes to immune cell recruitment and inflammation (7). Moreover, accumulation of egg antigens in the lungs of Sch-PAH patients was not found (8), which could suggest rapid clearance of the antigens in the initial stages of the pathology. Although pathogen-associated molecular pattern (PAMP)-induced EC activation contributes to the immune response, the effect of *S. mansoni* PAMPs and antigens on the pulmonary endothelium remains unclear.

Pulmonary ECs express high levels of the anti-inflammatory and endoprotective proteins Cav-1 and BMPR2, which are critical for regulating several processes, including cell survival and endothelial nitric oxide synthase (eNOS) function. These processes occur mostly via Cav-1 phosphorylation at the tyrosine 14 residue (pY14) (9–11), which can be induced by bacterial-derived PAMPs, such as lipopolysaccharide (LPS) exposure (12). However, if persistent, Cav-1 phosphorylation can result in Cav-1 depletion and inflammation, in part by shedding extracellular vesicles into the circulation (13). PAMPs stimulate immunity and host defense by activating pattern recognition receptors (PRRs) (14). Among PRRs, Toll-like receptor 4 (TLR4) plays a significant role in schistosomiasis by activating signaling in the immune system and ECs (15, 16). Interestingly, *TLR4^−/−^* mice do not develop PH (17), suggesting that this receptor is important during disease development. Activation of TLR4/CD14 leads to the activation of the NF-κB signaling pathway, but it also induces pY14-Cav-1 (14). Interestingly, our data indicate that *S. mansoni* egg antigens also increased pY14-Cav-1 expression and depletion in lung ECs. However, it is unclear whether *S. mansoni* antigens require TLR4/CD14-mediated pY14-Cav-1 for extracellular vesicle shedding.

Several biological processes such as apoptosis resistance and endothelial-to-mesenchymal transition have been described in PAH (18–20). All these processes contribute to the idea that chronically injured endothelium leads to the remodeling of the lung vasculature over time. Although the initiating events that promote alterations in healthy endothelium remain incompletely understood, it is possible that chronic exposure to pathogens induces the selective survival of an abnormal lung EC phenotype. In line with this hypothesis, we have observed a marked decrease in the expression levels of anti-inflammatory Cav-1 and BMPR2 within the lung vascular endothelium, which appear to be instrumental for the expansion of an abnormal EC phenotype evident in PAH. Additionally, our research data suggest that by regulating apoptosis-associated signaling pathways, there is potential to prevent cell death in Sch-PAH by imprinting an EC memory of survival. Although the specific mechanisms by which reduction in Cav-1 and BMPR2 expression have not been fully elucidated, our investigations underscore the pivotal role of both innate and acquired microorganisms in the pathophysiological cascade that underlines the disease.

## 2 Materials and methods

### 2.1 Animal model of Sch-PAH and lipopolysaccharide-induced acute lung vascular injury

The Sch-PAH mouse model was induced either by exposure to percutaneous infection using approximately 80 cercaria and animals used after 60 days of infection, or by murine intraperitoneal (IP) sensitization using 240 *S. mansoni* eggs/g body weight followed by intravenous (IV) injection of 175 eggs/g body weight after two weeks (21, 22). The specific model is highlighted in each figure legend (i.e., cercariae or IP/IV Eggs). Tail vein injections were performed in 2.5% isoflurane-anesthetized mice. The depth of anesthesia was monitored based on the lack of response to toe pinch. The mouse tail was submerged in water at 37°C or warmed with a heating lamp held approximately 20 cm away, avoiding overheating or burning, for 20–30 s for dilation of veins following injection of the eggs diluted in 100 µl of phosphate buffer solution (PBS; 30G needle). Mice were monitored daily, and after 7 days, the animals were anesthetized using ketamine/xylazine (K/X at 100 and 10 mg/kg body weight; IP) for subsequent analysis. After the procedure, mice were euthanized via cervical dislocation. Alternatively, C57BL6 mice (Jackson Laboratory, Bar Harbor, ME) were nebulized with saline or *Escherichia coli* LPS (10 mg, 1 h daily for up to 4 days) (13). Strain- and age-matched mice were used as approved by the Institutional Animal Care and Use Committee.

### 2.2 Hemodynamics and right ventricular hypertrophy assessment

After the animals underwent general anesthesia using K/X, as described above, the surgical area was disinfected using 70% alcohol, and a small skin incision was made to access the jugular vein. Then, a Millar Mikro-Tip catheter transducer (model PVR-1030) was carefully inserted into the right ventricle (RV) via *the* jugular vein to measure the RV systolic pressure (RVSP). RVSP was calculated using an MPVS-300 system connected to a PowerLab A/D converter (AD Instruments, Colorado Springs, CO, USA). After recording, mice were ventilated, and approximately 1 mL of blood was collected using 3.8% sodium citrate-treated syringes via vena cava puncture. The animals were then exsanguinated, and the remaining blood within the lungs was cleared by pump perfusion of 5 mL of cold PBS via a cannula placed in the RV. After complete perfusion, the lung lobes were either freshly removed, snap-frozen in liquid nitrogen, or carefully inflated with a 4% paraformaldehyde (PFA) solution for posterior histological analysis. Freshly isolated hearts were dissected to evaluate of RVH using the Fulton index (RV/left ventricle + septum weight ratio) (3).

### 2.3 Endothelial cell-specific Cav-1 deletion

Male and female 8–12 weeks old *End.SCL-cre^ERT2^*;*Rosa^mt/mg^* and *End.SCL-cre^ERT2^;Rosa^mt/mg^;Cav-1^fl/fl^* mice were IP injected with corn oil (vehicle) or tamoxifen for 5 consecutive days (1 mg/day) to induce cre-mediated recombination and EC-Cav-1 deletion (14 days after the last injection) (23). Briefly, after tamoxifen-induced recombination, ECs became green in both mouse genotypes (i.e., *End.SCL-cre^ERT2^;Rosa^mt/mg^* and *End.SCL-cre^ERT2^;Rosa^mt/mg^;Cav-1^fl/fl^*); however, in the group expressing *Cav1^fl/fl^* mice, ECs also lost Cav-1 expression (Cre-Lox recombination). Genetic recombination was confirmed by eGFP-positive vasculature via fluorescence imaging of the tail snips of anesthetized mice. Non-recombined cells remained positive for TdTomato (red fluorescence). The animals were then infected, as described above. Murine lung ECs (MLECs) were freshly isolated from collagenase-digested lung tissue for analysis, and cre recombinase-induced endothelial mEGFP fluorescent expression (green) in place of the mTomato (red) and Cav-1 deletion validated in all animal strains.

### 2.4 *S. mansoni* life cycle and egg collection

All studies with snails and cercariae were performed in a biological safety laboratory-2 (BSL-2) at Rush University Medical Center, with infected *Biomphalaria glabrata* snails obtained from the Schistosome Resource Center, Biomedical Research Institute (NIH-NIAID contract HHSN272201000005I). Between 7 and 8 weeks post-infection, the mice were euthanized with Nembutal in a buffered heparin solution, and eggs were collected from liver homogenates by serial filtration using autoclaved meshes inside the hood. The eggs were counted, resuspended in sterile PBS for fresh use, or stored at −80°C for subsequent injection in mice.

### 2.5 Human microvascular ECs from lungs and pulmonary artery ECs

Primary HMVEC-L and HPAEC (fourth passage) were obtained from Lonza (Cat No. CC-2527 and CC-2530, respectively) and maintained in endothelial basal medium-2 (EBM-2) supplemented with endothelial growth medium (EGM-2) SingleQuots™ Supplements (CC-4176; HPAEC) or MV SingleQuots (CC-4147; HMVEC-L) plus heat-inactivated fetal bovine serum up to 10% at 37°C and 5% CO_2_ until they reached 90%–100% confluence. Confluent cells were treated with *S. mansoni egg antigen* p40 (Sm-p40; Biomatik; Cat #RPC20108) for short-term signaling pathway analysis (0 min, 15 min, 30 min, 60 min, 120 min, and 240 min) or long-term analysis (24 h and 48 h). Cell morphology was assessed daily using brightfield contrast microscopy, and cell lysates were used for protein quantification. Cells were used up to the maximum level in the seventh passage.

### 2.6 Extracellular vesicle isolation and counting

Differential centrifugation and filtration methods were adapted to isolate extracellular vesicles (mainly microvesicles and small apoptotic bodies) from murine plasma, as previously described (3). Briefly, 3 mL of cell culture supernatant and 250 μL of plasma from the mice were centrifuged twice for 20 min (1,500×*g* at 4°C). The platelet-free plasma was centrifuged at 16,100×*g* for 20 min at 4°C. After centrifugation, the pellet was diluted in double-filtered PBS to determine its size and concentration by Nanoparticle Tracking Analysis (NanoSight; Malvern Analytical). The number of cells was used for normalization.

### 2.7 SEAP assay

The SEAP assay was performed using HEK-Blue™ hTLR4 cells grown in a medium containing Invitrogen HEK-Blue Selection (Cat #hb-sel), Invitrogen Normocin (Cat. #anti-nr-1), 50 mL Gibco fetal bovine serum (FBS; Cat #26140079), and 5 mL Pen Strep 10× (Gibco; Cat #15140-122) in 450 mL Corning DMEM with L-glutamate (Cat #10-013-cu). Cells were grown to 90% confluence and then split using trypsin-EDTA 1× and 10^5^ cells/well were plated in a 96 well-plate overnight. The cells were then pre-incubated for 1 h with 4 ng/mL anti-CD14 and 4 ng/mL anti-TLR4 (Invitrogen; Cat #mabg-htlr4), followed by exposure to 10 µg/mL LPS (Sigma; Cat No: L2630-25MG) and 1,000 ng/mL Sm-p40 (Biomatik; Cat #CP220360). All the conditions were diluted in complete medium to obtain the desired concentration. Cells were also transfected with 4 µg of GFP control and Y14F Cav-1 cDNA using Lipofectamine 3000 transfection reagent (Invitrogen; Cat #L-3000-015) in Gibco Opti-Mem (Cat #31985-070), before treatment. Plates were then measured at 24 h and 48 h using SeraCare Blue Phos Microwell phosphatase substrate system solutions A and B (Cat #5120-0061) diluted 1:1 with 1× PBS, and 100 μl/well was added and incubated 15 min before plate reading at 630 nm (Accuris Smartreader 96).

### 2.8 Sample preparation and Western blot

Frozen lung tissue was fully homogenized using a cold radioimmunoprecipitation assay (RIPA) buffer containing 1% protease and 0.1% phosphatase inhibitor cocktail. After 20 min of incubation at 4°C, the homogenates were centrifuged at 12,000*×g* (20 min at 4°C), and the supernatant was collected for protein measurement. A standard bicinchoninic acid (BCA) protein assay was used to determine the protein concentration in each lung sample by colorimetry using a microplate reader (Benchmark Scientific MR9600-T SmartReader™ 96 Plate). Then, 10 μg–30 μg of lung tissue lysate was diluted in Laemmli Sample Buffer (4×) with β-mercaptoethanol and boiled for 10 min at 95°C. Before loading the samples on a gradient SDS-PAGE gel (8%–12%), tubes were centrifuged for 2.5 min at 16,200*×g*. After running the samples, proteins were transferred to nitrocellulose membranes, and the transfer was assessed by the absence of ladder markers in the gel, simultaneously, to detect a continuous gradient of Ponceau Rouge staining over the membranes. Membranes were then washed with TBS-Tween 1× for 5 min twice and blocked using 5% milk or BSA (according to antibody datasheet instructions) for 1 h at room temperature, followed by primary antibody incubation (overnight at 4°C or 2 h–3 h at 37°C). After washing (2 min × 5 min; 1 min × 15 min), the membranes were incubated for 1 h with the specific secondary HRP-conjugated antibody, washed again, and detected using an ECL kit (Amersham, Piscataway, NJ). Membranes were scanned with Li-Cor Odyssey CLx (Lincoln, NE), and data were analyzed and normalized to β-actin or GAPDH loading controls using ImageJ software (https://imagej.nih.gov/ij/).

### 2.9 Immunohistochemistry and vascular remodeling

PFA-fixed, paraffin-embedded lung sections (5 μm) were used to evaluate protein expression (using primary antibodies against α-SMA, CD31, and Cav-1) and for histological analysis. Samples were deparaffinized by serial exposure to xylene and ethanol, and then high temperature was used to promote antigen retrieval (5 min–10 min in a pressurized container using sodium citrate as a buffer). For IHC, lung sections were blocked using 10% goat serum or 5% BSA diluted in PBS (1 h, room temperature), followed by overnight incubation with the primary antibody at 4°C (in a humidified chamber). After washing, the slides were incubated with secondary antibodies, washed again, and mounted using mounting medium containing DAPI. Fluorescent images were obtained using an LSM880 confocal microscope (Carl Zeiss MicroImaging, Inc.). Four fluorescent images were obtained from randomized peripheral lung segments in each sample to quantify Cav-1 expression. In addition, histological analysis of the microvessel area and thickness was performed using Masson’s trichrome-stained sections. After staining, slides containing one or two sections were scanned using an Aperio brightfield automated microscope slide scanner (×40; Leica Aperio AT2). Digitalized images were used to determine the microvessel area and thickness (μm) in 10–20 microvessels/animal, i.e., vessels with a diameter smaller than 100 μm, using ImageScope software 12.4.6 (Leica Biosystems). Briefly, microvessel wall thickness was obtained using the ImageScope ruler tool to measure the length across the thickest part of the vessel segment, defined as the space between the peripheral outer wall of the vasculature and the luminal boundary. The microvessel wall area was collected by tracing the contours of the peripheral outer wall and luminal boundary to obtain the total area and luminal area, respectively, and calculated by subtracting the luminal area from the total area of the microvessels (3).

### 2.10 Apoptosis assay by flow cytometry

HMVEC-L seeded in 6-well plates were treated with 1,000 ng/mL Sm-p40 for 24 h and 48 h, or 200 nM Staurosporine (STS; Tocris Cat No. 1285) for 18 h. The cells were then washed with PBS and gently detached using trypsin-EDTA 1× (Cat No. 15400-054; Gibco). Then, 5 × 10^6^ cells were incubated with APC Annexin V Apoptosis Detection kit with PI, according to the manufacturer’s protocol (Cat No. 640932; Biolegend). Data were acquired using Gallius (Beckman Coulter, USA), and the apoptotic cells were quantified in GFP-positive cells using Kaluza 2.2 (Beckman Coulter, USA).

### 2.11 *In situ* apoptosis assay

DNA cleavage was determined using terminal deoxynucleotide transferase (TdT)-mediated dUTP nick-end labeling (TUNEL) assays using an *in situ* cell death detection kit (ab206386; Abcam; Massachusetts, USA) according to the manufacturer’s instructions. The ratio of TUNEL positive to total cells (apoptotic index) was measured within the lung microvasculature (vessels >100 μm).

#### 2.11.1 Whole metagenomic analysis

Two fecal pellets and lung tissue from control and *S. mansoni* egg-stimulated mice were used for the metagenomic analysis. Samples from each animal were transferred to barcode collection tubes containing DNA stabilization buffer to ensure reproducibility and traceability and analyzed by automatic whole genome sequencing (WGS), which captures the DNA sequences of all microbes in a sample (Transnetyx, Cordova, USA). Briefly, after the DNA extraction, DNA samples were sequenced on both Illumina and ONT sequencing platforms. The raw data were obtained and analyzed by One Codex analysis software *via* alignment with the most recent One Codex Database (https://www.onecodex.com/, latest access date: 24 July 2023). This database is based on both public and private sources. Low-quality or mislabeled records were removed using a combination of automated and manual approaches. Then, using the One Codex analysis system, we obtained Shannon, Simpson, and Chao indices to compare microbiota richness and evenness in the samples (i.e., α diversity). Metagenomic classification as relative abundance was used to analyze the gut microbiome, whereas Readcounts_with_Children was used to compare the lung microbiome and analyze when the readcount was greater than 30K reads (egg/PBS alone baseline). In addition, each sample was individually analyzed at the phylum level using the percentage of classified reads using the most recent database updates and the most frequent top four phylum were plotted using GraphPad Prism v10. The remaining reads at the Phylum level were included as “Others” and represent less than 30% of the readcount in the lung and less than 15% readcount in the gut microbiome. Finally, the current One Codex Database consists of ~127K complete microbial genomes, including 73K bacterial, 49K viral, 1,817K fungal, 1,975K archaeal, and 201 protozoan genomes (127,034 including the host). Human and mouse genome are included to screen out host reads (24, 25). FASTQ files used in this study were analyzed using the One Codex Database and deposited in the NCBI Sequence Read Archive (SRA) under the BioProject ID PRJNA999692.

#### 2.11.2 Reagents and antibodies

Rabbit polyclonal anti-α-SMA was purchased from Abcam (Boston, MA, USA). Rabbit polyclonal anti-GAPDH antibody was acquired from Santa Cruz Biotechnology (Santa Cruz, CA, USA). Mouse monoclonal anti-total or phosphorylated eNOS, β-actin, and rabbit polyclonal anti-Cav-1 antibodies were purchased from BD PharMingen (San Jose, CA, USA). Alexa Fluor 488- and 555-conjugated goat anti-mouse and anti-rabbit IgG antibodies were purchased from Life Technologies (Grand Island, NY, USA). Anti-mouse and anti-rabbit HRP-conjugated IgG were purchased from Cell Signaling Technology (Danvers, MA, USA) and Kierkegaard & Perry Laboratories (Gaithersburg, MD, USA), respectively. RIPA buffer, protease and phosphatase inhibitor cocktail, collagenase type I, PFA, sodium citrate, heparin, and sucrose were purchased from SIGMA Chemical, Co. (St. Louis, MO, USA). Mounting media with DAPI (VectaShield) was obtained from Vector Laboratories (Burlingame, CA, USA). The batches 03614, 03577, and 03228 from Sm-p40 were acquired from Biomatik (Wilmington, Delaware, USA). Stock solutions were prepared in 100% dimethyl sulfoxide (DMSO) or sterile PBS and diluted daily in sterile PBS or cell medium for *in vitro* treatment. The highest final concentration of the solvent was 0.1% (v/v) and did not affect the experiments. PCR primers were purchased from Integrated DNA Technologies, Inc. (IDT; Coralville, Iowa, USA).

#### 2.11.3 Statistics and scientific rigor

Data were analyzed using the One Codex Cloud Platform and GraphPad Prism v10 (GraphPad, La Jolla, CA, USA). Normally distributed data are presented as arithmetic mean ± Standard Error of the Mean (SEM). The Shapiro–Wilk test were used to determine the normality of the data, and the Brown–Forsythe or F-test was used to assess the equality of variances. Parametric or nonparametric tests were then performed accordingly. Parametric statistical analysis was performed using unpaired Student’s t-test between two groups. One-way or two-way ANOVA followed by *post hoc* analysis (Bonferroni or Tukey Multiple Comparison tests) was used to analyze differences between more than two groups. Nonparametric analysis was performed using the Mann–Whitney U test. We used Shannon, Simpson, and Chao indices to compare the microbiota richness and evenness of the samples (i.e., α diversity). Relative abundance was used to analyze the gut microbiome, while the Readcount with Children was used to compare the lung microbiome (analyzed when greater than 1% of the total sequencing data). All tests were two-sided, and statistical significance was set at P <0.05.

## 3 Results

### 3.1 *S. mansoni* promoted depletion of lung endothelial Cav-1 expression in an animal model of Sch-PAH

**Figure 1:**
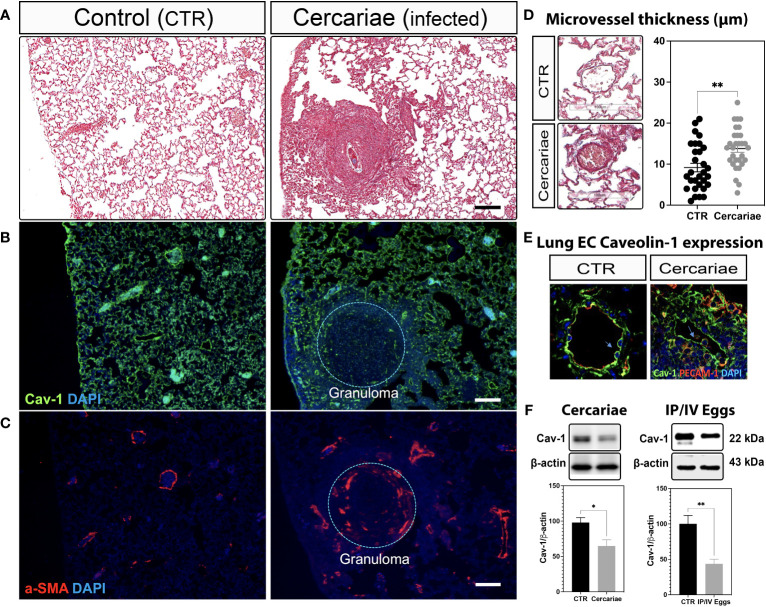
Lung Caveolin-1 expression is reduced in an animal model of *S. mansoni*-associated pulmonary hypertension. Lung sections from uninfected control (CTR) and *Schistosoma mansoni* percutaneously infected mice (~80 cercariae/animal) showing: Masson’s trichrome staining **(A, D)**, Caveolin-1 (Cav-1 **(B, E)**) in green and alpha-smooth muscle actin expression (α-SMA; **C**) or PECAM-1 **(E)** in red. Scale bar: 100 µm. The nuclei were stained with DAPI (blue). Microvessel thickness was quantified using ImageScope, and the data represent 10 vessels/animal **(D)**. Lung Cav-1 expression was quantified in percutaneously infected mice **(E)**. Total lung Cav-1 expression quantification in percutaneous cercariae infection **(F)** or *S. mansoni*-stimulated mice (intraperitoneal/intravenous eggs (IP/IV Eggs)) **(F)**. Normally distributed data were analyzed using unpaired Student’s t-test (n = 4–8 animals/group; *P <0.05; **P <0.01).

Recently, we showed that lung ECs from patients with IPAH are deficient in endothelial Cav-1 expression due to shedding of Cav-1-containing extracellular vesicles into the plasma (3, 26). These observations were further supported by the specific deletion and reconstitution of EC-Cav-1 in a mouse model of hypoxia-induced PH (3). Similarly, our data revealed that EC-Cav-1 deficiency also occurs within the remodeled and inflamed pulmonary area of animals chronically infected with *S. mansoni*, either percutaneously or stimulated with pathogenic eggs. However, outside the granuloma, EC-Cav-1 levels remained unchanged (**Figures 1A-F**; **Supplementary Figures 1A,B**). Taken together, these data indicate that an *S. mansoni* egg-associated mechanism might be sufficient to reduce the expression of the anti-inflammatory protein Cav-1 within the murine lung vasculature.

### 3.2 *S. mansoni* egg stimulus induced Cav-1 phosphorylation, BMPR2 depletion, and cell death in the lungs

**Figure 2:**
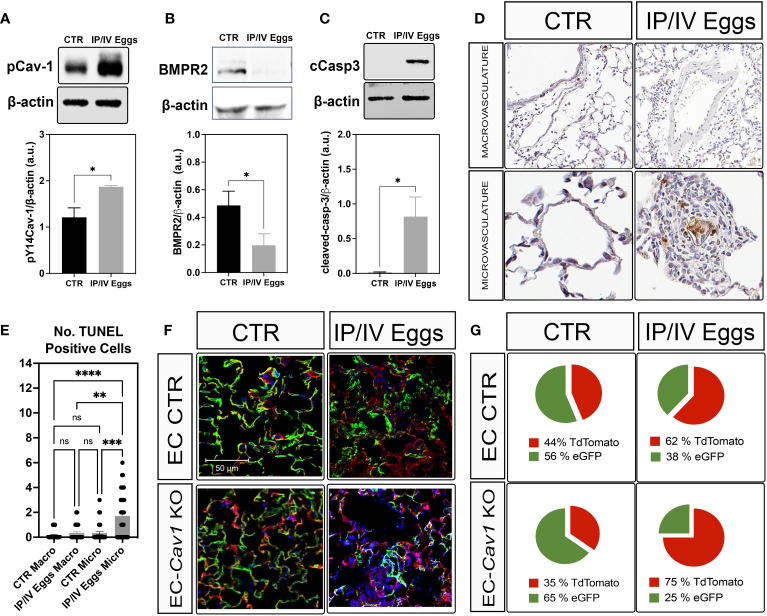
*S. mansoni* eggs induced pY14-Cav-1 and BMPR2 depletion, contributing to apoptosis within the lung microvasculature. **(A, B)** Lung phosphorylated Cav-1 at residue Y14 (pCav-1; A), BMPR2 **(B)**, and cleaved-Caspase-3 (cCasp3; **C**) expression in control (CTR) and *S. mansoni* egg- (IP/IV Eggs) stimulated mice. **(D, E)** *In situ* apoptosis by TUNEL staining of the lung macrovasculature (macro: >100 µm) and microvasculature (micro: <100 µm). **(F, G)** Lung sections from endothelial cell control (EC CTR) and IP/IV egg-stimulated *EndSclCreER^T2^;Rosa^mt/mg^* (control) +/− *Cav1^fl/fl^* (EC-Cav1 KO) mice showing the percentage of endothelial eGFP+ cells (green). Other non-recombined lung cells remained positive for tdTomato (red). Data were analyzed by Student’s t-test and Kruskal–Wallis (n = 4–8 animals per group; ns, non-significant; *P <0.05; **P <0.01; ***P <0.001; ****P <0.0001).

pY14-Cav-1 contributes to its deficiency and the survival of a Cav1^low^;BMPR2^low^ apoptosis-resistant EC phenotype in IPAH (3, 26, 27). Similarly, the lungs from infected mice displayed hyperphosphorylation of Cav-1 and reduced Cav-1 and BMPR2 expression (**Figures 1F**, **2A,B**). Cleaved-caspase-3 and TUNEL analyses revealed an increase in apoptosis within the lungs and remodeled microvasculature of *S. mansoni* egg-stimulated animals compared to the controls (**Figures 2C-E**). Further analysis of the dependence of Cav-1 expression on lung EC survival in Sch-PAH using our recently generated conditional lineage tracing EC-specific *Cav1^−/−^* mice (**Supplementary Figures 2A–C**) indicated that EC-Cav-1 deletion had no significant impact on the overall % of eGFP+ cells at baseline (**Figures 2F, G**), with the EC control (i.e., green ECs expressing Cav-1) and EC-*Cav1^−/−^* displaying 56% and 65% of GFP, respectively. Upon stimulation, eGFP+ cells were reduced in both groups, with a greater effect in EC-*Cav1^−/−^* mice (**Figures 2F, G**). Taken together, these data suggest that eggs and their antigenic molecules may contribute to the dysfunction and death of lung ECs observed in PAH.

### 3.3 Immunodominant *S. mansoni* egg antigen-induced transient pY14-Cav-1 and mild depletion of Cav-1

**Figure 3:**
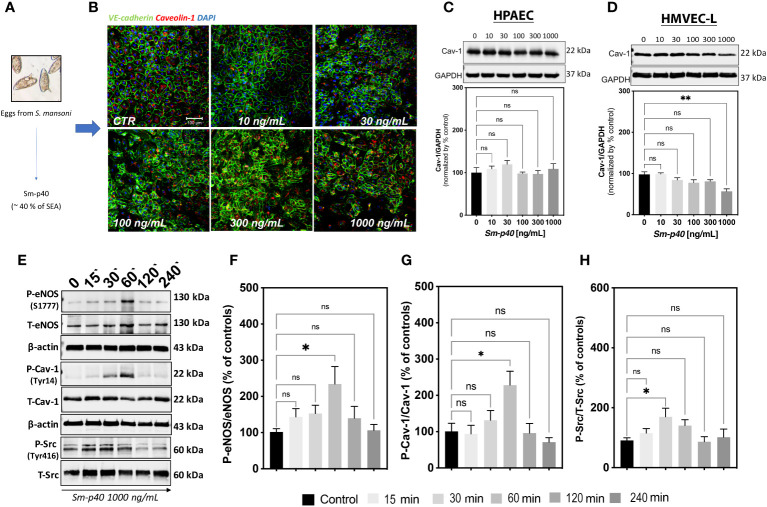
*S. mansoni* p40 activates lung microvascular endothelial cells leading to mild depletion of Cav-1. **(A)** *S. mansoni* p40 (Sm-p40) antigen accounts for 40% of the total egg antigen (SEA). **(B, C)** Sm-p40-treated (10 to 1,000 ng/ml) and untreated control human pulmonary artery endothelial cells (HPAECs; 3rd–7th passage) were used to evaluate of the Cav-1 and VE-cadherin expression (48 h). **(D-H)** Sm-p40 antigen-treated (10 to 1,000 ng/ml) and untreated control human lung microvascular lung endothelial cells (HMVEC-L; 3rd–7th passage) were used to measure the total and phosphorylated (P) expression of endothelial nitric oxide synthase (eNOS), Caveolin-1 (Cav-1), and Src. Data were analyzed by one-way ANOVA followed by the *post hoc* Dunnett test for statistical comparisons of normally distributed data (n = three different cultures and three Sm-p40 batches; *P <0.05; **P <0.01; ns, non-significant).

*S. mansoni* eggs secrete several antigenic molecules, with the Sm-p40 described as the immunodominant antigen capable of inducing a Th1-associated proinflammatory response (28). Thus, to test the effect of the Sm-p40 antigen on lung EC function and Cav-1 expression, we exposed HPAECs and HMVEC-L to 0 ng/ml–1,000 ng/ml Sm-p40 for up to 48 h. At the maximum time point (*i.e.*, 48 h), 1000 ng/ml Sm-p40 induced a slight reduction in VE-cadherin junctional stability with no effect on total Cav-1 expression in HPAECs (**Figures 3A-C**). In contrast, 48 h of exposure to the same concentration of Sm-p40 culminated in mild but significant depletion of Cav-1 expression, with no difference in total VE-cadherin expression in HMVEC-L (**Figure 3D**; **Supplementary Figures 3A,B**). In the short-term, Sm-p40 induced Src-associated phosphorylation of Cav-1 at the tyrosine-14 residue and eNOS at the serine-1177 (**Figures 3E-H**), indicative of activation of microvascular lung ECs.

### 3.4 Sm-p40 activates lung endothelial TLR4/CD14 signaling but does not induce endothelial apoptosis

**Figure 4:**
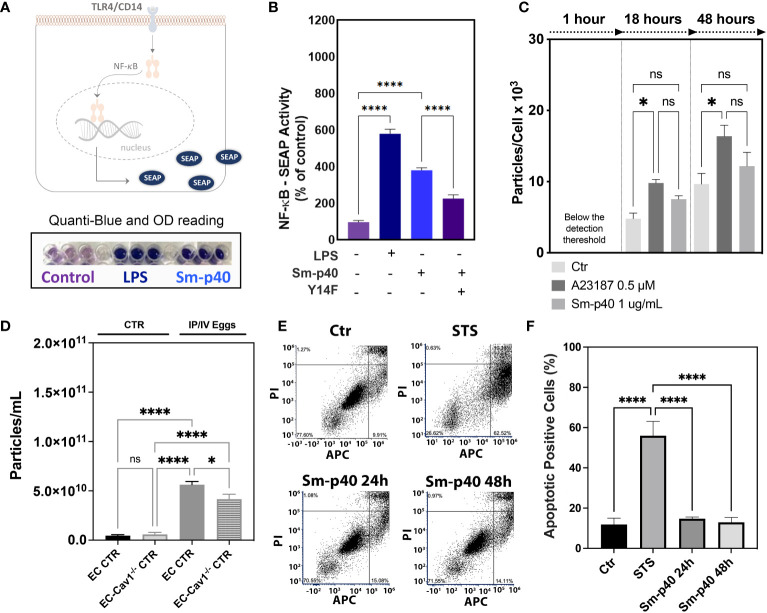
*S. mansoni* p40 activates TLR4 but fails to induce lung endothelial vesicle shedding or apoptosis. **(A, B)** Secreted alkaline phosphatase (SEAP) activity was measured in the supernatant of HEK-Blue™ hTLR4 cells 48 h after treatment with 1,000 ng/ml *S. mansoni* egg antigen p40 (Sm-p40) and lipopolysaccharide (LPS). Alternatively, cells were transfected with mutant Cav1Y14F and control cDNA. **(C)** Untreated HMVEC-L, A23187, and Sm-p40-treated cells were used to quantify extracellular vesicles. **(D)** Plasma from uninfected controls (CTR and endothelial cell CTR) and intraperitoneal/intravenous eggs (IP/IV Eggs) stimulated *EndSclCreER^T2^;Rosa^mt/mg^* (control) +/− *Cav1^fl/fl^* (ECKO) mice were used to quantify extracellular vesicles. **(E, F)** Flow cytometry dot plot and graphical quantification of the apoptotic positive % of control and Sm-p40 or Staurosporine (STS)-treated HMVEC-L for 24 h and 48 h. Data were analyzed by one-way ANOVA followed by the *post hoc* Dunnett test for normally distributed data (n = three mice/group and three different cultures; ns, non-significant; *P <0.05; ****P <0.0001).

Increased pCav-1 levels in lung ECs have been reported to be TLR4/CD14-dependent, culminating in Cav-1 depletion *via* shedding of extracellular vesicles and endothelial injury (12, 13). Similarly, Sm-p40-mediated pCav-1 was at least in part, dependent on the TLR4/CD14-mediated signaling pathway (**Figures 4A, B**) but failed to induce significant shedding of extracellular vesicles *in vitro*, compared to the positive control, the calcium ionophore A23187 (**Figure 4C**). *Ex vivo* data revealed an increase in the concentration of circulating extracellular vesicles concentration in the plasma of stimulated mice compared to controls, and this effect was reduced by 26% by conditional deletion of endothelial Cav-1 expression (**Figure 4D**). Finally, either 24 h or 48 h exposure to Sm-p40 was unable to induce apoptosis in HMVEC-L compared to the positive control staurosporine *in vitro* (**Figures 4E, F**), suggesting that *S. mansoni* egg-derived p40 activates TLR4/CD14/py14-Cav-1 signaling pathway, but additional host factors might be required for lung endothelial injury and expansion of the abnormal vascular phenotype observed in PAH.

### 3.5 *S. mansoni* egg stimulus disrupted the host’s gut–lung microbiota contributing to severe murine PH

**Figure 5:**
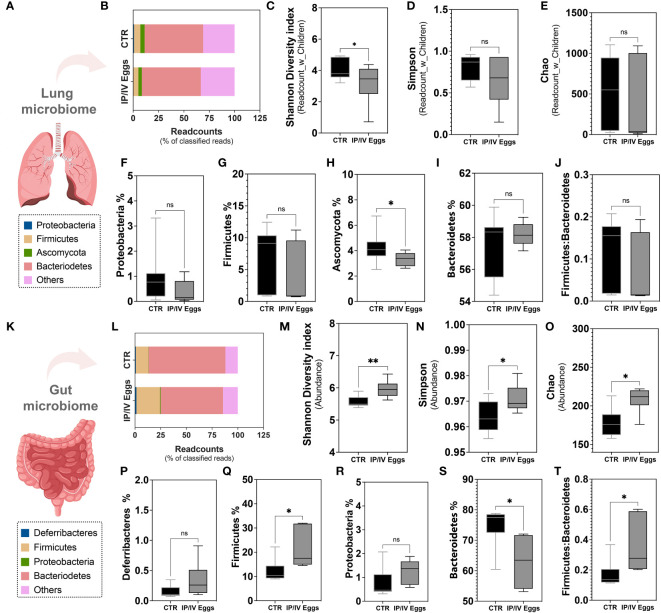
*S. mansoni* egg stimulus induced gut–lung microbiome dysbiosis in mice model of PH. Lung tissue **(A–J)** and fecal pellets **(K–T)** from uninfected control (CTR; black bars) and *S. mansoni* intraperitoneal/intravenous eggs (IP/IV Eggs) stimulated mice (dark gray bars) were used for whole genome sequencing and metagenomic analysis using the One Codex Database (access date: 07/24/2023). **(B, P)** Readcount % of top 4 frequent Phyla: Proteobacteria (**F**, lung) or Deferribacteres (**P**, gut) (blue), Firmicutes (**G, Q**, yellow), Ascomycota (**H**, lung) or Proteobacteria (**R**, gut) (green), Bacteroidetes (**S**, red), and others (pink). **(C–E)** α-diversity (expressed as Readcount_with_Children (lung) and Abundance (gut): Shannon diversity index **(C, M)**, Simpson index **(D, N)**, and Chao index (Observed taxa at the species level; **E, O**). **(J, T)** Firmicutes : Bacteroidetes ratios in the lungs and gut. Data were analyzed using Student’s t-test and Mann–Whitney test (n = 6–11 mice/group; ns, non-significant; *P <0.05; **P <0.01).

Metagenomic analysis revealed that *S. mansoni* egg stimulation disrupted the lung microbiome, leading to a significant reduction in the Phylum Ascomycota compared to controls (**Figures 5A-J**). *S. mansoni* eggs also reduced α-diversity in the lungs, whereas the ratio Firmicutes : Bacteroidetes remained similar between groups (**Figures 5C-J**). No difference was observed in Simpson and Chao indices (**Figures 5D, E**). In the gut, the presence of eggs increased α-diversity and did not alter the relative abundance of Deferribacteres and Proteobacteria, but significantly increased the Firmicutes : Bacteroidetes ratio, indicative of gut dysbiosis (**Figures 5K-T**). Taken together, these findings indicate that *S. mansoni* egg exposure disrupts the gut–lung host microbiota.

**Figure 6:**
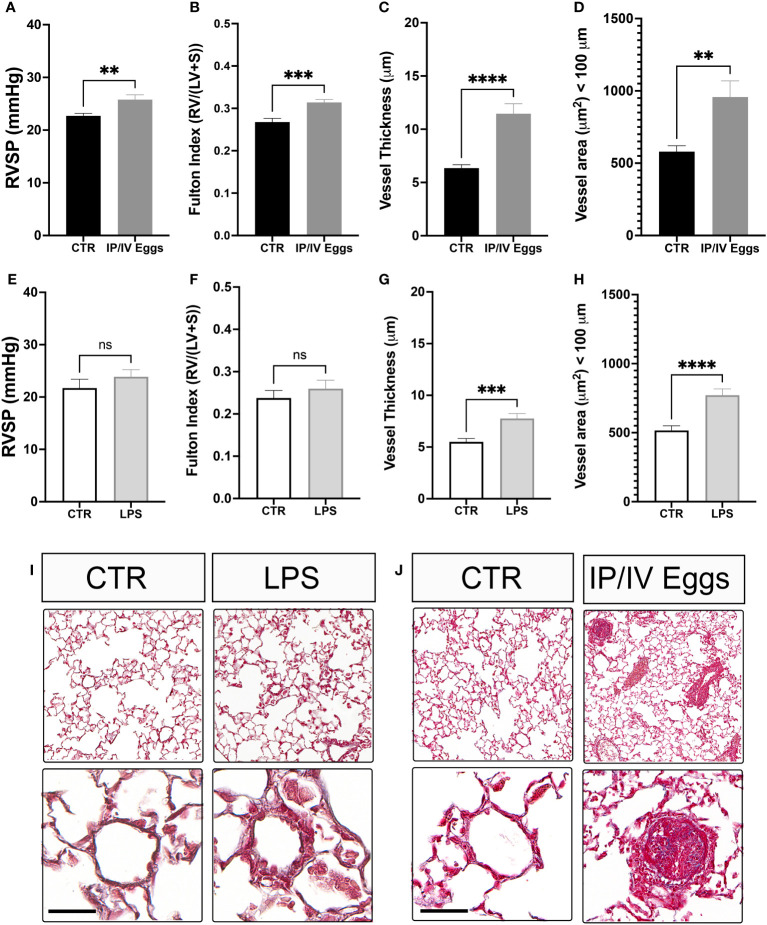
*Schistosoma mansoni* egg stimulation induces severe pulmonary hypertension in mice. Control (CTR), lipopolysaccharide (LPS)-treated **(E–I)**, and *S. mansoni* intraperitoneal/intravenous egg (IP/IV Egg) stimulated **(A–D, J)** mice were used to evaluate of the right ventricular systolic pressure (RVSP), right ventricular hypertrophy (RVH; Fulton index), pulmonary microvascular thickness and area using *Masson’s trichome* staining. LPS control (white bars), LPS-treated (light gray bars), uninfected CTR (black bars), and *S. mansoni* IP/IV egg-stimulated mice (dark gray bars). Data were analyzed using Student’s t-test (n = 5–10 mice/group; ns, non-significant; **P <0.01; ***P <0.001; ****P <0.0001).

**Figure 7:**
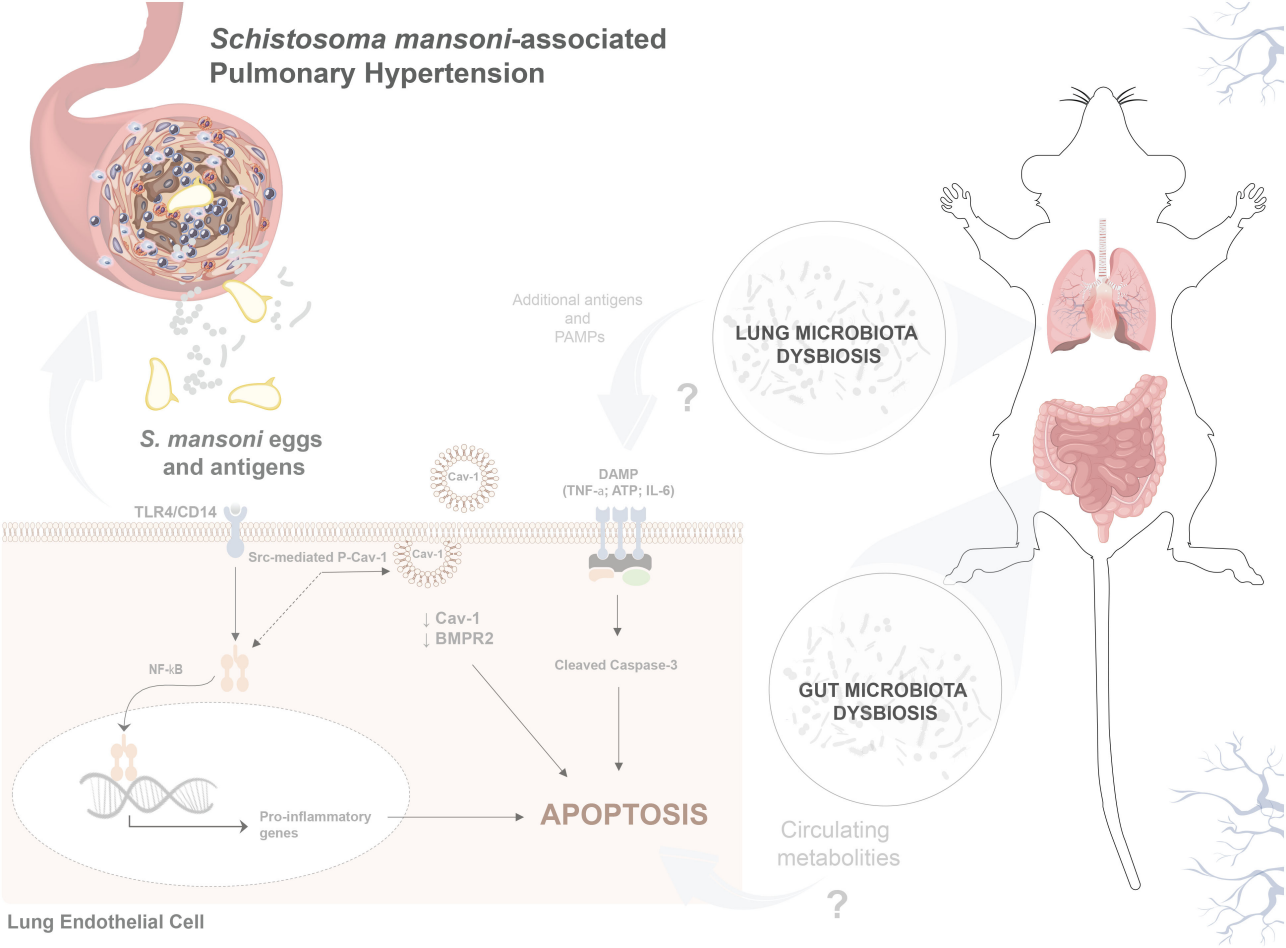
*S. mansoni* egg stimulation induces gut–lung microbiome dysbiosis and severe pulmonary hypertension in mice. Sch-PAH is a life-threatening complication of chronic *S. mansoni* infection that leads to heart failure and eventual death. Abnormal ECs that are resistant to apoptosis, contribute to disease progression. Reduction of Cav-1 and BMPR2 expression shifts EC quiescence towards an abnormal death-resistant cell phenotype. Gut–lung microbiome disruption, *S. mansoni* eggs, and antigens play crucial roles in promoting abnormal EC expansion. *In vitro*, the immunodominant Sm-p40 antigen induced TLR4/CD14-mediated Cav-1 phosphorylation culminating in Cav-1 depletion. *In vivo*, infection induces microvascular cell death and severe pulmonary vascular remodeling, leading to elevated right ventricular systolic pressure and hypertrophy, which are characteristics of PAH.

Indeed, *S. mansoni* egg stimulation promoted severe vascular remodeling and increased RVSP and RVH (**Figures 6A-D, J**). Although TLR4 activation plays a role in the development of murine PH, the classical bacterial-derived TLR4 agonist LPS alone did not induce murine PH (**Figures 6E–I**), suggesting that the combination of *S. mansoni* eggs, their antigens, and a disrupted host microbiota plays a key role in the full establishment of Sch-PAH (**Figure 7**).

## 4 Discussion

Lung endothelial Cav-1 participates in several processes, such as the regulation of receptors and enzymes that are fundamental for EC quiescence and physiology, including BMPR2 and eNOS. Previously, we observed that pathogen-derived products, specifically bacterial LPS, reduced endothelial Cav-1 expression and canonical BMPR2-P-SMAD1/5/8 signaling, leading to exacerbated TGF-β-mediated signaling within the lungs and contributing to inflammatory and reversible microvascular remodeling (13). Indeed, BMPR2 deficiency is known to promote cell death, endothelial-to-mesenchymal transition (endoMT), and abnormal proliferation of pulmonary vascular cells, including ECs, which contribute to the severe pulmonary vascular remodeling observed in several subgroups of PAH (29–33). Moreover, it has been shown that heterozygous *Bmpr2^+/−^* mice have increased *S. mansoni* egg translocation in the lungs via enlarged hepatic sinusoids (34), and *Bmpr2^+R899X^* loss-of-function mutant mice develop “spontaneous” PH, reinforcing the crucial role of this signaling pathway in the development of PAH, including the infectious disease (35).

Apoptosis-resistant cell growth and endoMT have been extensively described in PAH (19, 20, 36). Despite how the process is entitled, it has become substantially evident that chronically injured endothelium leads to remodeling of the lung vasculature over time. Although the initiating events that promote alterations in healthy endothelium remain unclear, it is possible that chronic exposure to pathogens and their PAMPs induces EC dysfunction and selective survival of an abnormal EC phenotype. Previously, we showed that mesentery ECs from *S. mansoni*-infected mice maintained epigenetic memory of the infection *in vitro* (21, 37). This memory is characterized by dysfunctional purinergic signaling, reduced nitric oxide levels, and elevated EC–leukocyte interactions. However, whether and how lung ECs generate inflammatory memory in pathogen-induced vascular disease is still unknown. EC injury in IPAH and animal models of hypoxia-induced PH is associated with Cav-1 phosphorylation and depletion via shedding of extracellular vesicles into the circulation (13). Despite previous studies’ findings on EC- Cav-1/BMPR2 depletion as an important switch towards endothelial dysfunction (21, 31, 35), more specific information about what triggers the process remains unclear. Here, we observed that pathogens can contribute to EC-Cav-1/BMPR2 depletion and initiate Sch-PAH. Specifically, our data also indicate that *S. mansoni* egg antigen p40 increases pY14-Cav-1 in lung microvascular ECs, culminating in its depletion. Further molecular studies are needed to investigate how the Cav-1/BMPR2 interaction modulates signaling in ECs.

*S. mansoni* eggs and soluble egg antigens (i.e., SEA) have been described as key players during the inflammatory process by modulating the Th1/Th2 polarization observed in the disease (38), but little is known about their effects on lung EC function. Among SEA, the Sm-p40 antigen is a 40 kDa glycoprotein reported as the immunodominant egg antigen (28). Sm-p40 is also one of the most abundant antigenic molecules, accounting for approximately 40% of the total SEA, and is capable of inducing a strong Th1-associated response (39). In contrast, other antigenic molecules in SEA, including omega-1 (40), contribute to inducing a potent Th2-associated response by elevating IL-13 expression (41). During schistosomiasis, Th2-derived IL-4/IL-13 contributes to the activation of macrophage-derived TGF-β, leading to severe pulmonary vascular remodeling (2). While an exacerbated Th1 response contributes to cell injury, Th2-derived IL-13 also promotes the migration of abnormal lung ECs (42), indicating that pathogen-induced Th1/2-endothelial communication is critical for the expansion of an abnormal EC phenotype. Finally, based on previous and current findings, it is undeniable that egg-mediated Th2-related events, including recruitment of Th2 CD4+ lymphocytes, increased thrombospondin synthesis, and macrophage-derived TGF-β secretion and activation, are critical biological events for progression towards a severely remodeled and inflamed pulmonary vasculature. However, it is also evident that Th1-related tissue damage is an important initial step in the inflammatory cascade.

It is not novel that pathogens and their antigenic molecules activate receptors on professional immune cells or ECs, contributing to the inflammatory response. However, the mechanism by which this interaction modulates immunity is unclear. Our data revealed that Sm-p40 antigen activates TLR4/CD14, contributing to Cav-1 phosphorylation and depletion *in vitro*. Moreover, *in vivo*, we observed that infection significantly reduced Cav-1 and BMPR2 expression and promoted significant pulmonary vascular injury and remodeling. Although only eggs are sufficient to induce significant remodeling in the lung vasculature, our data indicated that altered gut–lung microbiota also participates in this process. More than 30 years ago, evidence revealed that a milder schistosomiasis-associated inflammatory response is observed in germ-free mice (43), supporting our observations that intrinsic microbiota disruption contributes to the inflammatory process underlying Sch-PAH. Indeed, percutaneous *S. mansoni* infection reveals pathogen transposition from the gut mesentery into the lungs (4). Gastrointestinal damage and gut dysbiosis have been previously reported (44, 45), with infection with female and male *S. mansoni* contributes to a higher murine susceptibility to colitis and preclinical inflammatory bowel disease, but to the best of our knowledge, the role of gut dysbiosis and lung microbiome in the development of Sch-PAH has been elusive until now.

Recently, several studies have supported the idea of translocating microorganisms and their metabolites from the gut environment into the lungs (46–49). Similarly to our data, Callejo et al. (47), described significant changes in the gut microbiome in hypoxia and SU5416-induced PH model in rats, including increased Firmicutes/Bacteroidetes ratio compared to controls. Another recent study by Moutsoglou et al. also provided translational evidence for gut dysbiosis and circulating metabolites in patients with PAH (49, 50), reinforcing the role of disruption of the human gut microbiota disruption in the development of PAH. In fact, knowledge of the mechanisms underlying the microbiome and its role in health and disease remains recent, especially when evaluating the lung environment. To the best of our knowledge, our data provide the first evidence of lung microbiome dysbiosis in Sch-PAH, indicating that reduced α-diversity, especially the reduced percentage of the phylum Ascomycota, may play an important role in the disease. The microbiome is a reservoir for antimicrobial resistance genes, and the phylum Ascomycota comprises several anti-inflammatory and antibiotic-producing microorganisms that are relevant for human health. However, we recognize that the limited percentage of microorganisms in the lungs indicates the urgent need for further investigation and novel technical approaches to reveal the specific details of microorganisms, their PRRs, and associated signaling pathways in health and disease. For example, a deeper mycobiome analysis may reveal important information about other non-bacterial microorganisms that contribute to the development of PH. Thus, although our study highlights important associations between schistosomiasis, gut–lung microbiome dysbiosis, and the development of PH in an animal model, establishing a direct mechanistic link remains challenging. A comprehensive approach, involving manipulating the microbiome and using human-derived samples would likely provide deeper insights into these intricate cause/effect relationships. Moreover, among the PRRs relevant to pathogen recognition and inflammatory response, *TLR4^−/−^* mice have been reported to be resistant to the development of hypoxia-induced PH in mice; however, our previous and current data indicate that LPS-induced TLR4 activation alone promoted only reversible remodeling of the pulmonary vasculature (13) with no significant increase in RVSP or RVH in mice. On the other hand, exposure to *S. mansoni* eggs and their antigens promoted severe remodeling of the pulmonary vasculature and elevated RVSP and RVH, indicating that the parasite eggs contain crucial molecules with a high potential to uncover a novel signaling pathway and trigger molecular targets to understand the onset and progression of PAH and for the development of future target therapeutic approaches to cure or ameliorate the morbidity associated with the disease.

## Data availability statement

The datasets presented in this study can be found in online repositories. The names of the repository/repositories and accession number(s) can be found below: PRJNA999692 (SRA).

## Ethics statement

Ethical approval was not required for the studies on humans in accordance with the local legislation and institutional requirements because only commercially available established cell lines were used. The animal study was approved by UIC Institutional Animal Care and Use Committee. The study was conducted in accordance with the local legislation and institutional requirements.

## Author contributions

YM: Data curation, Formal Analysis, Investigation, Methodology, Software, Validation, Visualization, Writing – original draft, Writing – review & editing. EV: Data curation, Formal Analysis, Investigation, Methodology, Software, Validation, Visualization, Writing – original draft, Writing – review & editing. SA: Investigation, Methodology, Resources, Writing – review & editing. DW: Investigation, Methodology, Resources, Visualization, Writing – review & editing. JS: Investigation, Methodology, Visualization, Writing – review & editing. CS: Formal Analysis, Investigation, Methodology, Resources, Visualization, Writing – review & editing. SL: Investigation, Methodology, Visualization, Writing – review & editing. SO: Conceptualization, Data curation, Formal Analysis, Funding acquisition, Investigation, Methodology, Project administration, Resources, Software, Supervision, Validation, Visualization, Writing – original draft, Writing – review & editing.

## References

[1] Knafl D Gerges C King CH Humbert M Bustinduy AL. Schistosomiasis-associated pulmonary arterial hypertension: a systematic review. Eur Respir Rev (2020) 29:190089. doi: 10.1183/16000617.0089-2019 32024722 PMC9488905

[2] Kumar R Mickael C Kassa B Gebreab L Robinson JC Koyanagi DE. TGF-β activation by bone marrow-derived thrombospondin-1 causes Schistosoma- and hypoxia-induced pulmonary hypertension. Nat Commun (2017) 8:15494. doi: 10.1038/ncomms15494 28555642 PMC5459967

[3] Oliveira SDS Chen J Castellon M Mao M Raj JU Comhair S. M. R. Injury-induced shedding of extracellular vesicles depletes endothelial cells of cav-1 (Caveolin-1) and enables TGF-β (Transforming growth factor-β)-dependent pulmonary arterial hypertension. ATVB (2019) 39:1191–202. doi: 10.1161/ATVBAHA.118.312038 PMC7297129 30943774

[4] Oliveira SD. Insights on the gut-mesentery-lung axis in pulmonary arterial hypertension: A poorly investigated crossroad. Arterioscler Thromb Vasc Biol (2022) 42:516–26. doi: 10.1161/ATVBAHA.121.316236 PMC9050827 35296152

[5] O’Dwyer DN Dickson RP Moore BB. The lung microbiome, immunity, and the pathogenesis of chronic lung disease. J Immunol (2016) 196:4839–47. doi: 10.4049/jimmunol.1600279 PMC4894335 27260767

[6] Oswald IP Eltoum I Wynn TA Schwartz B Caspar P Paulin D. Endothelial cells are activated by cytokine treatment to kill an intravascular parasite, Schistosoma mansoni, through the production of nitric oxide. Proc Natl Acad Sci (1994) 91:999–1003. doi: 10.1073/pnas.91.3.999 7508126 PMC521441

[7] Costain AH MacDonald AS Smits HH. Schistosome egg migration: mechanisms, pathogenesis and host immune responses. Front Immunol (2018) 9. doi: 10.3389/fimmu.2018.03042 PMC6306409 30619372

[8] Graham BB Chabon J Bandeira A Espinheira L Butrous G Tuder RM. Significant intrapulmonary schistosoma egg antigens are not present in schistosomiasis-associated pulmonary hypertension. Pulm Circ (2011) 1:456–61. doi: 10.4103/2045-8932.93544 PMC3329075 22530100

[9] Chen ZDS Oliveira S Zimnicka AM Jiang Y Sharma T Chen S. Reciprocal regulation of eNOS and caveolin-1 functions in endothelial cells. Mol Biol Cell (2018) 29:1190–202. doi: 10.1091/mbc.E17-01-0049. mbc.E17-01-0049. PMC5935069 29563255

[10] Oliveira SDS Minshall RD. Caveolin and endothelial NO signaling. Curr Top Membr (2018), 82:257–79. doi: 10.1016/bs.ctm.2018.09.004 30360781

[11] Chen Z Bakhshi FR Shajahan AN Sharma T Mao M Trane A. Nitric oxide-dependent Src activation and resultant caveolin-1 phosphorylation promote eNOS/caveolin-1 binding and eNOS inhibition. Mol Biol Cell (2012) 23:1388–98. doi: 10.1091/mbc.e11-09-0811 PMC3315804 22323292

[12] Jiao H Zhang Y Yan Z Wang Z-G Liu G Minshall RD. Caveolin-1 Tyr14 phosphorylation induces interaction with TLR4 in endothelial cells and mediates MyD88-dependent signaling and sepsis-induced lung inflammation. J Immunol (2013) 191:6191–9. doi: 10.4049/jimmunol.1300873 PMC3874812 24244013

[13] Oliveira SDS Castellon M Chen J Bonini MG Gu X Elliott MH. Inflammation-induced caveolin-1 and BMPRII depletion promotes endothelial dysfunction and TGF-β-driven pulmonary vascular remodeling. Am J Physiol Lung Cell Mol Physiol (2017) 312:L760–71. doi: 10.1152/ajplung.00484.2016 PMC5451590 28188225

[14] Fitzgerald KA Kagan JC. Toll-like receptors and the control of immunity. Cell (2020) 180:1044–66. doi: 10.1016/j.cell.2020.02.041 PMC9358771 32164908

[15] Kane CM Jung E Pearce EJ. Schistosoma mansoni egg antigen-mediated modulation of Toll-Like Receptor (TLR)-induced activation occurs independently of TLR2, TLR4, and MyD88. Infect Immun (2008) 76:5754–9. doi: 10.1128/IAI.00497-08 PMC2583582 18824534

[16] Ritter M Gross O Kays S Ruland J Nimmerjahn F Saijo S. Schistosoma mansoni triggers Dectin-2, which activates the Nlrp3 inflammasome and alters adaptive immune responses. Proc Natl Acad Sci (2010) 107:20459–64. doi: 10.1073/pnas.1010337107 PMC2996650 21059925

[17] Young KC Hussein SMA Dadiz R deMello D Devia C Hehre D. Toll-like receptor 4–deficient mice are resistant to chronic hypoxia-induced pulmonary hypertension. Exp Lung Res (2010) 36:111–9. doi: 10.3109/01902140903171610 20205596

[18] Cool CD Kuebler WM Bogaard HJ Spiekerkoetter E Nicolls MR Voelkel NF. The hallmarks of severe pulmonary arterial hypertension: the cancer hypothesis—ten years later. Am J Physiol Cell Mol Physiol (2020) 318:L1115–30. doi: 10.1152/ajplung.00476.2019 PMC9847334 32023082

[19] Tuder RM Groves B Badesch DB Voelkel NF. Exuberant endothelial cell growth and elements of inflammation are present in plexiform lesions of pulmonary hypertension. Am J Pathol (1994) 144:275–85. PMC1887146 7508683

[20] Sakao S Taraseviciene‐Stewart L Lee JD Wood K Cool CD Voelkel NF. Initial apoptosis is followed by increased proliferation of apoptosis-resistant endothelial cells. FASEB J (2005) 19:1178–80. doi: 10.1096/fj.04-3261fje 15897232

[21] Oliveira SDS Quintas LEM Amaral LS Noël F Farsky SH Silva CLM. Increased endothelial cell-leukocyte interaction in murine schistosomiasis: Possible priming of endothelial cells by the disease. PLoS One (2011) 6:e23547. doi: 10.1371/journal.pone.0023547 21853150 PMC3154496

[22] Graham BB Kumar R. Schistosomiasis and the pulmonary vasculature (2013 Grover Conference series). Pulm Circ (2014) 4:353–62. doi: 10.1086/675983 PMC4278594 25621148

[23] Muzumdar MD Tasic B Miyamichi K Li L Luo L. A global double-fluorescent Cre reporter mouse. genesis (2007) 45:593–605. doi: 10.1002/dvg.20335 17868096

[24] Yang C Sung J Long D Alghoul Z Merlin D. Prevention of ulcerative colitis by autologous metabolite transfer from colitogenic microbiota treated with lipid nanoparticles encapsulating an anti-inflammatory drug candidate. Pharmaceutics (2022) 14:1233. doi: 10.3390/pharmaceutics14061233 35745805 PMC9228491

[25] Mishkin N Ricart Arbona RJ Carrasco SE Lawton S Henderson KS Momtsios P. Reemergence of the murine bacterial pathogen chlamydia muridarum in research mouse colonies. Comp Med (2022) 72:230–42. doi: 10.30802/AALAS-CM-22-000045 PMC9413529 35803706

[26] Bakhshi FR Mao M Shajahan AN Piegeler T Chen Z Chernaya O. Nitrosation-dependent caveolin 1 phosphorylation, ubiquitination, and degradation and its association with idiopathic pulmonary arterial hypertension. Pulm Circ (2013) 3:816–30. doi: 10.1086/674753 PMC4070841 25006397

[27] Maniatis NA Shinin V Schraufnagel DE Okada S Vogel SM Malik AB. Increased pulmonary vascular resistance and defective pulmonary artery filling in caveolin-1^-/-^ mice. Am J Physiol Lung Cell Mol Physiol (2008) 294:L865–73. doi: 10.1152/ajplung.00079.2007 PMC9819781 18192592

[28] Finger E Finardi de Souza A Galante Coimbra T dos Santos D. Immunodominance of the Sm-p40 antigen contributes to the development of severe schistosomiasis mansoni (99.5). J Immunol (2011) 186:99.5–5.

[29] Soon E Crosby A Southwood M Yang P Tajsic T Toshner M. Bone morphogenetic protein receptor type II deficiency and increased inflammatory cytokine production: A gateway to pulmonary arterial hypertension. Am J Respir Crit Care Med (2015) 192:859–72. doi: 10.1164/rccm.201408-1509OC PMC4613895 26073741

[30] Hopper RK Moonen J-RAJ Diebold I Cao A Rhodes CJ Tojais NF. In pulmonary arterial hypertension, reduced BMPR2 promotes endothelial-to-mesenchymal transition *via* HMGA1 and its target slug. Circulation (2016) 133:1783–94. doi: 10.1161/CIRCULATIONAHA.115.020617 PMC4856565 27045138

[31] Oliveira SDS Chen J Castellon M Mao M Raj JU Comhair S. Injury-induced shedding of extracellular vesicles depletes endothelial cells of cav-1 (Caveolin-1) and enables TGF-β (Transforming growth factor-β)-dependent pulmonary arterial hypertension. Arterioscler Thromb Vasc Biol (2019) 39:1191–202. doi: 10.1161/ATVBAHA.118.312038 PMC7297129 30943774

[32] Rabinovitch M. Molecular pathogenesis of pulmonary arterial hypertension. Ther J Clin Investig (2012) 122:4306–13. doi: 10.1172/JCI60658 PMC3533531 23202738

[33] Masri FA Xu W Comhair SAA Asosingh K Koo M Vasanji A. Hyperproliferative apoptosis-resistant endothelial cells in idiopathic pulmonary arterial hypertension. Am J Physiol Lung Cell Mol Physiol (2007) 293:L548. doi: 10.1152/ajplung.00428.2006 17526595

[34] Crosby A Soon E Jones FM Southwood MR Haghighat L Toshner MR. Hepatic shunting of eggs and pulmonary vascular remodeling in bmpr2 ^+/–^ mice with schistosomiasis. Am J Respir Crit Care Med (2015) 192:1355–65. doi: 10.1164/rccm.201412-2262OC PMC4731697 26308618

[35] Erewele EO Castellon M Loya O Marshboom G Schwartz A Yerlioglu K. Hypoxia-induced pulmonary hypertension upregulates eNOS and TGF-β contributing to sex-linked differences in BMPR2 ^+/R899X^ mutant mice. Pulm Circ (2022) 12. doi: 10.1002/pul2.12163 PMC9722973 36484056

[36] Cool CD Kuebler WM Bogaard HJ Spiekerkoetter E Nicolls MR Voelkel NF. The Hallmarks of Severe Pulmonary Arterial Hypertension: The Cancer Hypothesis - Ten years later. Am J Physiol Cell Mol Physiol (2020) 318:L1115–30. doi: 10.1152/ajplung.00476.2019 PMC9847334 32023082

[37] Oliveira SDS Oliveira NF Meyer-Fernandes JR Savio LEB Ornelas FGI Ferreira ZS. Increased expression of NTPDases 2 and 3 in mesenteric endothelial cells during schistosomiasis favors leukocyte adhesion through P2Y1receptors. Vasc Pharmacol (2016) 82:66–72. doi: 10.1016/j.vph.2016.02.005 26924460

[38] Caldas IR Campi-Azevedo AC Oliveira LFA Silveira AMS Oliveira RC Gazzinelli G. Human schistosomiasis mansoni: Immune responses during acute and chronic phases of the infection. Acta Trop (2008) 108:109–17. doi: 10.1016/j.actatropica.2008.05.027 18577364

[39] Stadecker MJ Hernandez HJ. The immune response and immunopathology in infection with Schistosoma mansoni : a key role of major egg antigen Sm-p40. Parasite Immunol (1998) 20:217–21. doi: 10.1046/j.1365-3024.1998.00150.x 9651922

[40] Everts B Hussaarts L Driessen NN Meevissen MHJ Schramm G van der Ham AJ. Schistosome-derived omega-1 drives Th2 polarization by suppressing protein synthesis following internalization by the mannose receptor. J Exp Med (2012) 209:1753–67. doi: 10.1084/jem.20111381 22966004 PMC3457738

[41] Stadecker MJ Hernandez HJ Asahi H. The identification and characterization of new immunogenic egg components: implications for evaluation and control of the immunopathogenic T cell response in schistosomiasis. Mem Inst Oswaldo Cruz (2001) 96:29–33. doi: 10.1590/S0074-02762001000900004 11586423

[42] Takagi K Yamakuchi M Matsuyama T Kondo K Uchida A Misono S. IL-13 enhances mesenchymal transition of pulmonary artery endothelial cells *via* down-regulation of miR-424/503 in *vitro* . Cell Signal (2018) 42:270–80. doi: 10.1016/j.cellsig.2017.10.019 29102771

[43] Viera LQ Moraes-santos T. Schistosomiasis mansoni: evidence for a milder response in germfree mice. Rev Inst Med Trop Sao Paulo (1987) 29:37–42. doi: 10.1590/S0036-46651987000100006 3114864

[44] Holzscheiter M Layland LE Loffredo-Verde E Mair K Vogelmann R Langer R. Lack of host gut microbiota alters immune responses and intestinal granuloma formation during schistosomiasis. Clin Exp Immunol (2014) 175:246–57. doi: 10.1111/cei.12230 PMC3892416 24168057

[45] Floudas A Aviello G Schwartz C Jeffery IB O’Toole PW Fallon PG. Schistosoma mansoni worm infection regulates the intestinal microbiota and susceptibility to colitis. Infect Immun (2019) 87. doi: 10.1128/IAI.00275-19 PMC6652750 31138616

[46] Ranchoux B Bigorgne A Hautefort A Girerd B Sitbon O Montani D. Gut–lung connection in pulmonary arterial hypertension. Am J Respir Cell Mol Biol (2017) 56:402–5. doi: 10.1165/rcmb.2015-0404LE 28248132

[47] Callejo M Mondejar-Parreño G Barreira B Izquierdo-Garcia JL Morales-Cano D Esquivel-Ruiz S. Pulmonary arterial hypertension affects the rat gut microbiome. Sci Rep (2018) 8:9681. doi: 10.1038/s41598-018-27682-w 29946072 PMC6018770

[48] Kim S Rigatto K Gazzana MB Knorst MM Richards EM Pepine CJ. Altered gut microbiome profile in patients with pulmonary arterial hypertension. Hypertension (2020) 75:1063–71. doi: 10.1161/HYPERTENSIONAHA.119.14294 PMC7067661 32088998

[49] Moutsoglou DM Tatah J Prisco SZ Prins KW Staley C Lopez S. Pulmonary arteBrial hypertension patients have a proinflammatory gut microbiome and altered circulating microbial metabolites. Am J Respir Crit Care Med (2022) 207:740–56. doi: 10.1164/rccm.202203-0490OC PMC10037487 36343281

[50] Oliveira SD. Cardiopulmonary pathogenic networks: unveiling the gut–lung microbiome axis in pulmonary arterial hypertension. Am J Respir Crit Care Med (2023) 207:655–7. doi: 10.1164/rccm.202211-2126ED PMC10037478 36476165

