# Supplementary material for: Schistosomiasis-associated pulmonary hypertension unveils disrupted murine gut–lung microbiome and reduced endoprotective Caveolin-1/BMPR2 expression
Source: Front Immunol. 2023 Oct 16;14:1254762. doi: 10.3389/fimmu.2023.1254762 (PMC10613683; doi:10.3389/fimmu.2023.1254762)
Supplement: Supplementary file 1 [file DataSheet_1.docx]

Supplementary Material

## Supplementary Figures


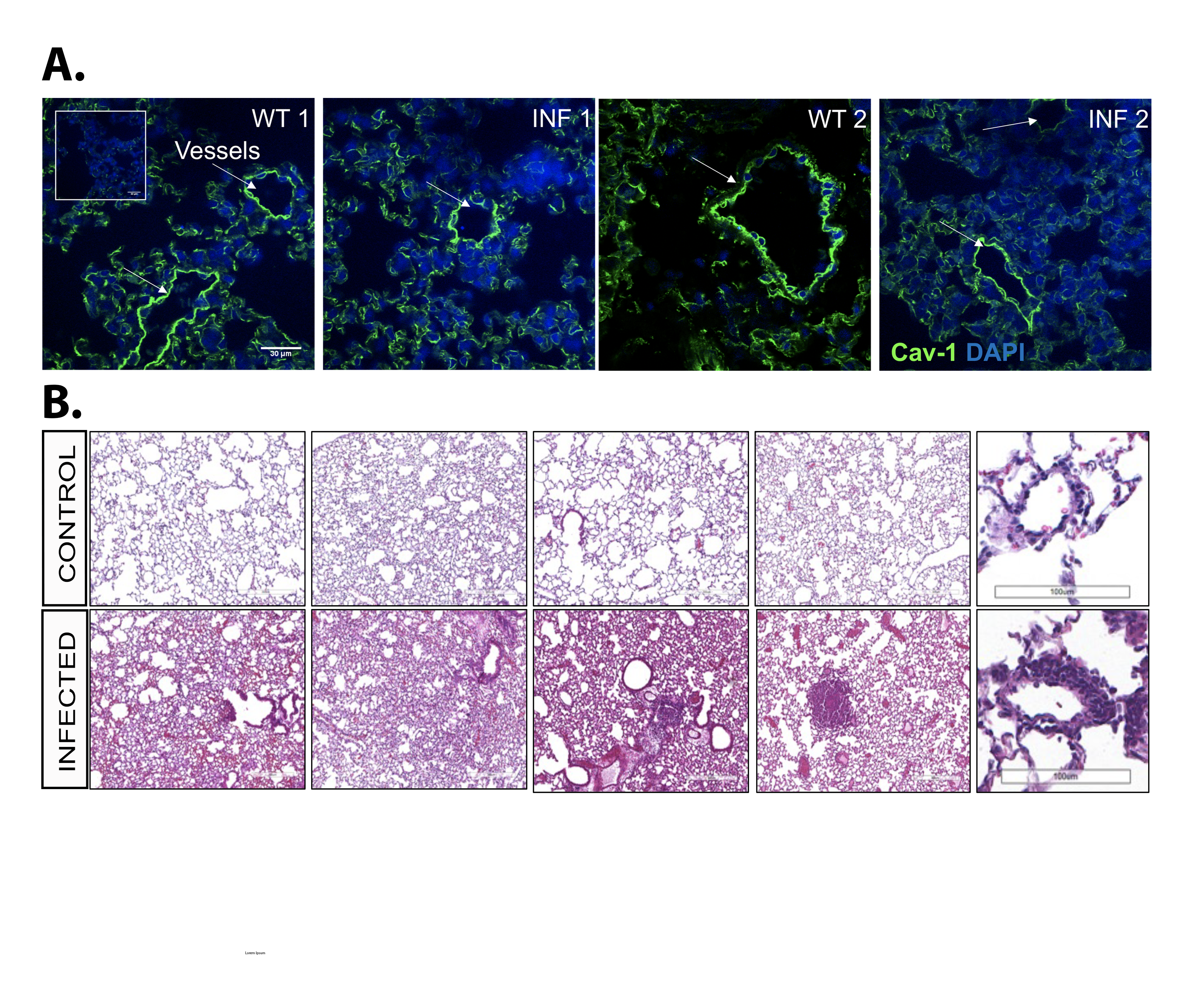


**Supplementary Figure 1.** Cav-1 expression is not altered in the vessels outside the granuloma area. **A.** Lung sections from normal control and Schistosoma mansoni-infected mice showing Cav-1 (green) expression. Scale bar: 50 µm. (n = 3 animals/group). **B.** Hematoxylin and eosin staining showing inflamed lungs upon percutaneous S mansoni infection. Inset: microvasculature. n = 4 mice/group.

**
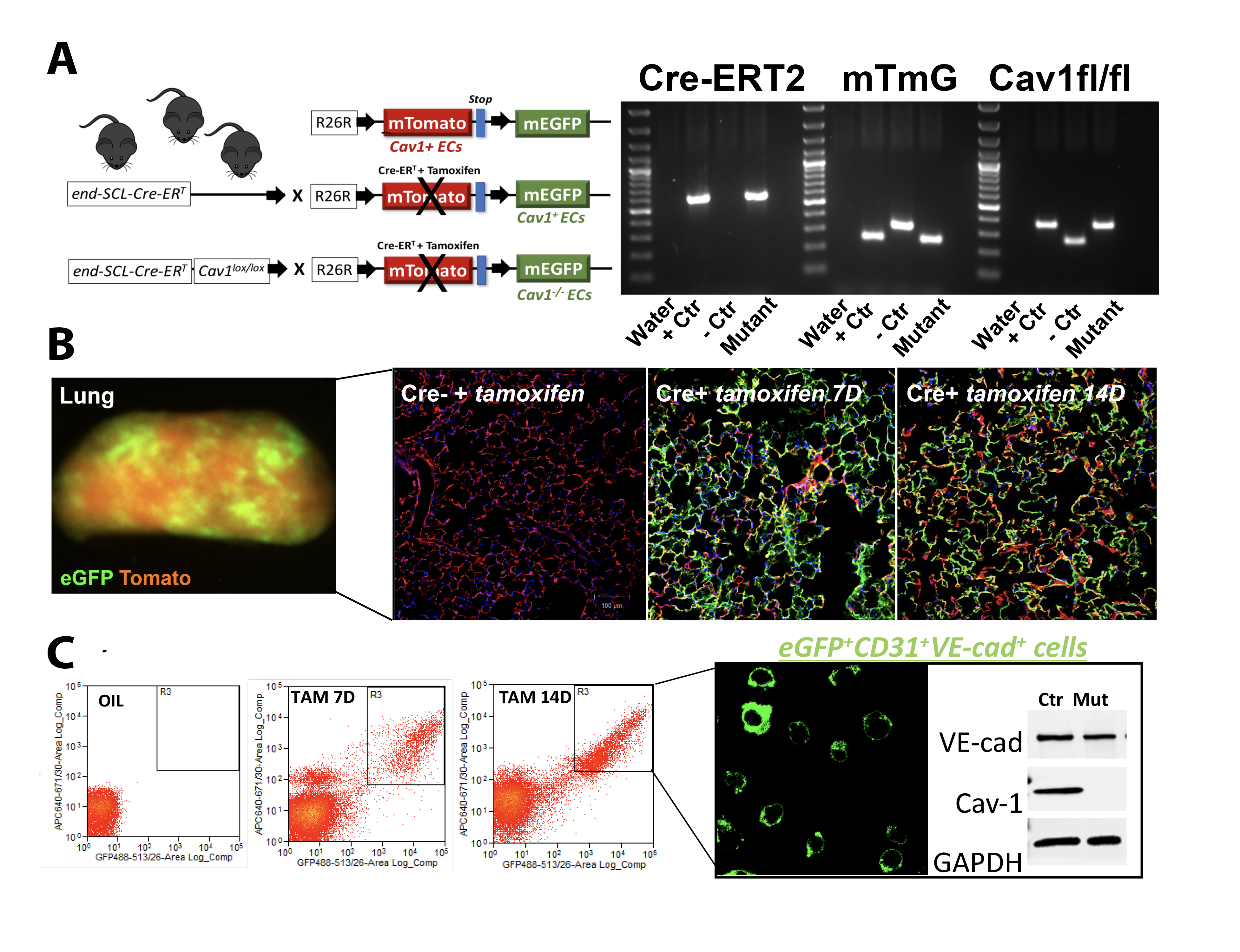
Supplementary Figure 2.** Endothelial-specific *Cav1^-/-^* and lineage reporter mouse. **A.** Generation of *EndSclCre.ER^T2^;Rosa^mt/mg^* and *EndSclCre^ERT2^;Rosa^mt/mg^* and +/- *Cav1^fl/fl^* allele. Inset: PCR blot showing *End.Scl.Cre.ER^T2^*, *Rosa^mt/mg^*, and *Cav1^fl/fl^*, respectively. Lanes were loaded with water, positive control (+ ctr), negative (-) ctr, and DNA from mutant (mut) *Cdh5-Cre.ER^T2^;Rosa^mt/mg^;Cav-1^fl/fl^* mice, respectively. **B.** Tamoxifen-induced cre recombination (endothelial cells in green (eGFP+); other lung cells in red (Tomato+). ***Inset:*** Lungs sections from cre- and cre+ (*EndSclCreER^T2^;Rosa^mt/mg^;Cav1^fl/fl^)* mutants 7 and 14 days after tamoxifen injections. **C.** Fresh-perfused lungs were used to isolate cells after tissue digestion with collagenase. After red blood cell lysis, a single cell suspension was blocked and stained with CD31-APC antibody. Cell sorting was used to isolate CD31+eGFP+ cells from tomato+ cells. ***Inset:*** The absence of “red cells” in the sorted cells is visible by confocal microscopy. Lysates were used to determine Cav-1 and VE-cadherin expression.


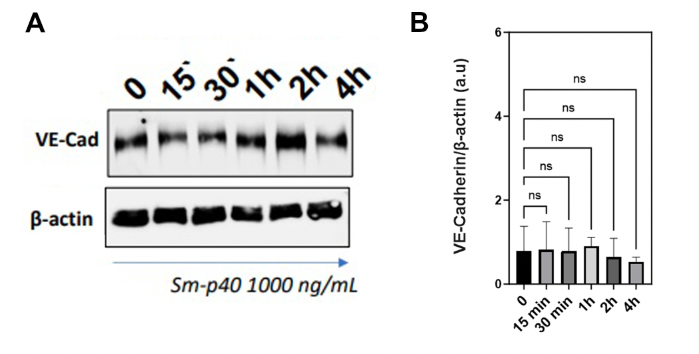


**Supplementary Figure 3. Sm-p40 alone does not change VE-cadherin expression in lung endothelial cells  (A-B).***S. mansoni* p40 antigen (Sm-p40)-treated (1000 ng/ml) and untreated human microvascular lung pulmonary endothelial cells (HMVEC-L; 3-7th passage) were used for quantification of VE-cadherin expression. Data were analyzed by one-way ANOVA followed by the post hoc Dunnet test for statistical comparisons in normally distributed data (n = 2 different cultures; ns = non-significant).
